# The effect of adaptive capacity on resilience to the COVID-19 pandemic: A cross-country analysis

**DOI:** 10.4102/jamba.v16i1.1697

**Published:** 2024-07-25

**Authors:** Asmita Khadka

**Affiliations:** 1 Graduate School of Public Administration, National Institute of Development Administration, Bangkok, Thailand

**Keywords:** adaptive capacity, disaster resilience, institutional quality, collaborative governance, social capital, biological hazards, disasters

## Abstract

The COVID-19 pandemic’s profound impacts on global health, driven by preparedness gaps and systemic risks, underscore the need to enhance societies’ ability to manage both predictable risks and uncertainties inherent in disasters. While disaster research emphasises risk management for predictable threats and adaptive capacity for unexpected challenges, there is a lack of empirical examination of the impact of adaptive capacity on disaster resilience. This study addresses this gap by identifying three key adaptive capacities – quality of institutions, collaborative governance, and social capital – and examining their effects on COVID-19 resilience outcomes, measured by the ability to reduce excess mortality. Analysing secondary data from 129 nations using partial least squares structural equation modelling, the research finds significant positive effects of institutional quality and social capital on resilience outcomes. Conversely, collaborative governance shows a significant negative association, suggesting potentially intricate impacts beyond initial expectations. The findings highlight the need to enhance institutional quality and social capital to address preparedness gaps and unexpected challenges posed by biological hazards such as COVID-19. Future research should explore collaborative governance using a disaggregated approach that considers the roles of different stakeholders in various disaster phases.

**Contribution:**

This study advances disaster research by presenting practical methodologies for operationalising adaptive capacities and empirically examining their effects on disaster resilience. For practitioners and policymakers, it highlights the need to adopt a long-term perspective in building disaster resilience, focussing on improving institutional quality and social capital to manage the uncertainties and complexities inherent in disaster scenarios effectively.

## Introduction

Societal approaches to handling disasters have witnessed significant transformations, evolving from the conventional emergency response approach to a comprehensive disaster risk management (DRM) framework, and subsequently placing a heightened emphasis on disaster resilience. Disaster resilience is defined as the capacity of a social system to withstand, absorb, and swiftly recover from a disaster event (Adger 2000; Alwang, Siegel & Jorgensen 2001; UNISDR 2009). The growing emphasis on disaster resilience stems from the recognition that certain risks are unpredictable, and uncertainty is an inherent aspect of society within our highly interconnected and complex world (Fekete, Hufschmidt & Kruse 2014). Unlike risk management, which addresses known risks, the resilience approach centres on improving a system’s ability to adapt and navigate the unexpected and continuously evolving in response to changing conditions (Fekete et al. 2014; Rosati, Touzinsky & Lillycrop 2015). Therefore, enhanced adaptability becomes a central aspect of a resilient system, particularly crucial when governments and societies face challenges in predicting the level of impacts and preparing and planning adequately for future crises, as illustrated by the coronavirus disease 2019 (COVID-19) pandemic.

The profound impacts of the COVID-19 pandemic on human lives, unexpectedly more pronounced in affluent regions, underscored the critical need for societies to be adaptive. Despite warnings about pandemic zoonoses, a lack of political attention created significant preparedness gap globally (Ibert et al. 2022), contributing to the devastating consequences of the pandemic. By 31 December 2023, the global impact reached over 774 million cases and 7 million deaths (WHO 2024), with widespread consequences across sectors, scales, and geographical boundaries. The impact has defied conventional expectations, particularly regarding the vulnerability and resilience of different economic strata. Despite past research suggesting that low-income countries are more prone to human impacts and high-income countries are susceptible to economic impacts (Rentschler 2013), the pandemic has inflicted a significant toll on human health in higher-income economies. The World Health Organization (WHO) data between January 2020 and December 2021 shows that lower-middle-income countries experienced the highest excess deaths per 100 000 populations, followed by high-income, upper-middle-income, and the lowest rates were observed in low-income countries (Msemburi et al. 2023). Regional analysis indicated that the European Region faced the highest excess deaths, closely followed by the Regions of Americas (Msemburi et al. 2023). However, the pandemic also showcased the adaptability of communities and societies in the face of unexpected events, but the varying extent of their adaptive capacity resulted in diverse outcomes (Ringsmuth et al. 2022). In the light of these observations, two crucial questions arise: What elements constitute a society’s adaptive capacity and do they significantly contribute to the overall resilience in the face of the COVID-19 pandemic?

In disaster research, two primary perspectives on adaptive capacity are recognised. One perspective focusses on the underlying capacities that facilitate adaptation to disaster risks on a larger scale, while the other examines hazard-specific adaptation strategies at smaller scales, such as the community level (Mortreux & Barnett 2017). The former perspective considers adaptive capacity the generic abilities that enhance disaster resilience by supporting planned responses to disaster risks and fostering creative solutions from social actors (Eakin, Lemos & Nelson 2014).

There are multiple gaps in studies employing this broader perspective of adaptive capacity. Firstly, as the resilience perspective gains prominence, its primary focus centres on climate-related and geophysical hazards, often neglecting exploration into biological hazards. Secondly, while scholars such as Berman, Quinn and Paavola (2012), Engle (2011), and Zukowski (2014) underscore the importance of examining disaster resilience in relation to adaptive capacity; its operationalisation is limited, with only a few researchers, such as Parsons et al. (2021), Tinch et al. (2015), and Barr, Fankhauser and Hamilton (2010), taking steps in this direction. Furthermore, a crucial need arises to distinguish adaptive capacity from coping capacity to enhance empirical analysis (Paloviita et al. 2017) and decision-making (Birkmann 2013). Although there are conceptual overlaps, coping capacity is generally perceived as addressing short-term needs through resources and abilities such as economic capacity and infrastructure for effective disaster response, while adaptive capacity is viewed as longer-term-oriented abilities that promote flexibility, adaptation, collaborative action, and transformation (Hulke & Diez 2020; Parsons et al. 2021; Tinch et al. 2015). Thus, adaptive capacity may influence how existing resources are leveraged (Berman et al. 2012), leading to varying outcomes even among economically advanced nations.

In the light of these considerations, this study aims to identify and operationalise adaptive capacities and investigate their roles in determining COVID-19 resilience outcomes.

## Theoretical background

### Concept of disaster resilience

The term ‘resilience’ stems from the Latin word ‘resilio’, denoting a system’s capacity to revert to its initial state after disruption (Klein, Nicholls & Thomalla 2003). The notion of disaster resilience originates from the ecological definition of resilience by Holling (1973), who characterises ecological resilience as a measure of a system’s persistence in the face of disruptions, emphasising the acceptance of large fluctuations to enhance endurance. Building on Holling’s definition, Adger (2000), Alwang et al. (2001), Cutter et al. (2008), and Manyena (2006) define disaster resilience as a system’s ability to resist, withstand, or recover quickly from shocks. In contrast to Holling’s ecological perspective that embraces large fluctuations, Wenger (2017) and Adger (2000) underscore human stability as crucial for disaster resilience.

The research diverges on whether resilience is assessed before or after a disaster. Ex-ante resilience measurements focus on quantifying attributes across social, economic, infrastructural, institutional, and environmental dimensions. Disaster resilience frameworks employing this approach, as seen in Mayunga (2007), Cutter et al. (2008), Parsons et al. (2016), and Kruk et al. (2017), often aim to create aggregate measures using various indicators as proxies for resilience levels. However, these frameworks primarily focus on quantifying disaster resilience at the community level, with limited applicability to the national level. Additionally, several proposed resilience indices lack empirical validation, a critical aspect for justifying their use in assessing and monitoring disaster resilience. Through a systematic review, Cai et al. (2018) found that only 10.3% of studies empirically validated their proposed resilience indices.

In contrast, ex post measurements shift focus to assessing damage, response, and recovery, effectively addressing validation issues. Some scholars measure disaster resilience solely in terms of the system’s ability to reduce disaster impact, as observed in the works of Mızrak and Çam (2022), while others, such as Rayamajhee, Bohara and Storr (2020), focus on the ability to recover quickly. In addition, scholars such as Lam et al. (2016), Lee (2019), and Hallegatte (2014) incorporate both impact reduction and quick recovery in their assessments.

### Adaptive capacity

Adaptive capacity is recognised as a crucial element for mitigating disaster vulnerability and enhancing resilience in conjunction with coping capacity. Although coping capacity is commonly emphasised in disaster risk studies and adaptive capacity in climate change studies, some research in both fields highlights the distinct and significant roles of both concepts.

Insights from Parsons et al. (2021), Welle et al. (2013), Dunford et al. (2015), and Tinch et al. (2015) indicate that adaptive capacity is oriented towards the long-term, involving capabilities that support adaptation to current or future disaster events. Parsons et al. (2016) define adaptive capacity as ‘the arrangements and processes that enable adjustment through learning, adaptation, and transformation’. Berkes (2007) argues that adaptation is not a predetermined consequence but a result of human agency, involving the influence of people, leaders, and institutions on the adaptive process. Adaptive capacity is commonly understood to encompass institutional and governance capacities, as well as social capital (Hulke & Diez 2020; Gupta et al. 2010; Keskitalo et al. 2011; Parsons et al. 2021; Pelling et al. 2008).

In assessing the role of adaptive capacity in disaster resilience, it is essential to distinguish between the quality of institutions, collaborative governance, and social capital. The quality of institutions pertains to the performance of government bodies (Barbier & Burgess 2021; Kasdan 2022). In contrast, collaborative governance involves government-led initiatives that engage both state and non-state actors in policymaking (Ansell & Gash 2008). Social capital, on the other hand, encompasses the relational dynamics within communities and societies (Ndlovu & Msimanga 2023; Straub et al. 2020). The mechanisms through which these capacities influence disaster response vary: for instance, during the COVID-19 pandemic, the quality of institutions influenced the stringency of public health policies (Wang 2022); formal collaborations among government bodies, intergovernmental organisations, and non-profit organisations significantly improved pandemic response planning and execution in low-income nations (Gooding et al. 2022) and social capital played a crucial role in ensuring compliance with government directives in many countries (Alfano 2022; Wu 2021). However, in capability-based disaster resilience measurement studies, indicators of collaborative governance are often incorporated into measures of institutional or governance capacity (e.g. Melore & Nel 2020; Parsons et al. 2016, 2021), which can obscure the distinction between the quality of government institutions and the extent of collaborative governance. Although non-state actors can participate in DRM efforts, government agencies are primarily responsible for leading these efforts (Hizbaron, Ruslanjari & Mardiatno 2021; Van Well et al. 2018). Additionally, disaster research on collaborative governance remains largely conceptual (Russell et al. 2021), leaving gaps in our understanding of how collaborative governance influences disaster resilience. Differentiating and understanding the distinct roles of government institutions, collaborative governance, and social capital is crucial for a nuanced understanding of adaptive capacity’s influence on disaster resilience, ultimately guiding policymakers in devising more effective resilience-building strategies.

### Theoretical framework: Adaptive capacity and resilience to COVID-19

Drawing from a literature review on the resilience of socio-ecological systems, primarily centred on disaster resilience research that includes studies on biological and other natural hazards, as well as relevant literature on climate change adaptation and adaptive governance, three specific capacities encompassing adaptive capacity have been identified. These capacities are hypothesised to be pivotal factors influencing a nation’s resilience to the COVID-19 pandemic, and corresponding hypotheses have been formulated for empirical examination.

#### Quality of institutions

The literature consistently highlights the significant role institutions and governance play in fostering disaster resilience. Institutional capacity stands out as a crucial component frequently employed in ex-ante disaster resilience indices (Yoon, Kang & Brody 2016). Effectively implementing policies to mitigate and address disaster risks, as well as enhancing the adaptiveness of individuals and communities, hinges on the pivotal role of institutions (Berman et al. 2012; Djalante et al. 2013; Oppenheim et al. 2019; Tiernan et al. 2019).

In empirical research, the evaluation of national-level institutional quality often relies on the World Bank’s World Governance Indicators (WGI), which assesses various dimensions of governance, including ‘government effectiveness, political stability, regulatory quality, control of corruption, rule of law, and voice and accountability’ (World Bank 2020b). In a study involving 14 Asian and Pacific nations, Taghizadeh-Hesary et al. (2021) discovered a significant positive impact of institutional quality, gauged by control of corruption, on reducing the harmful effects of natural hazards. In the context of COVID-19, Da Silva et al. (2023) found significant positive associations between various WGI indicators and decreased excess mortality. Similarly, Nabin, Chowdhury and Bhattacharya (2021) observed that countries with higher WGI levels exhibited higher COVID-19 vaccination rates and demonstrated greater control over the virus’s spread.

In light of the findings from the studies examining the relationship between the quality of institutions and disaster resilience outcomes, alongside indicators of institutional quality, the following research hypothesis can be formulated:

**Hypothesis 1:** Government effectiveness, political stability, regulatory quality, control of corruption, and rule of law reflect the quality of institutions, which exerts a significant positive effect on the COVID-19 resilience outcome.

(Note: In this study, the ‘voice and accountability’ indicator of the WGI, which corresponds to democracy level, is not considered an indicator of institutional quality. Instead, democracy level is regarded as one of the indicators of collaborative governance.)

#### Collaborative governance

Collaborative governance involves cooperation between state and non-state entities in decision-making and policy processes (Ansell & Gash 2008). Kapucu (2014) illuminates the aftermath of Hurricane Katrina, revealing a lack of coordination among government bodies and political conflicts in recovery efforts, and suggests that the complexity of recovery planning underscores the need for pre- and post-disaster collaboration among various stakeholders. Demiroz and Hu (2014) stress the critical role of civil society organisations in disaster risk reduction, mainly when government resources are insufficient. A systematic review by Fridell et al. (2020) on health system resilience underscores the significance of institutional features, including polycentric governance, collaboration across sectors, and accountable and transparent practices. Analysing South Korea’s response to COVID-19 through the adaptive governance lens, Kim et al. (2020) identify strengths such as decision-making grounded in evidence, a proactive stance in disaster response, the capacity to galvanise joint efforts among public health professionals and the empowerment of local governments. Non-governmental organisations (NGOs) played a crucial role in supporting South Korea’s response through close partnerships with local authorities and service providers, urging attention to disadvantaged communities (Choi 2020).

In discussions on COVID-19, Hegele and Schnabel (2021) advocate for a centralised governance model, while Yang (2020) highlights the significance of striking a balance between centralisation and decentralisation in the aftermath of the crisis. Nevertheless, decentralisation has been found to contribute to improved long-term health outcomes (Jiménez-Rubio 2011; Robalino, Picazo & Voetberg 2001), potentially reducing people’s vulnerability to infectious diseases such as COVID-19. Countries with a higher degree of decentralisation also exhibit fewer casualties from disaster events (Skidmore & Toya 2013). Cavalieri and Ferrante (2016) found that decentralisation not only facilitates the formulation of tailored policies but also enhances the accountability of local leaders, ultimately leading to improved health outcomes.

The literature on biological disasters underscores the criticality of a government’s capacity to form networks and cooperate with international stakeholders. Over recent decades, international donor agencies have assumed an increased role in pandemic management, providing financial support to low-income nations and implementing cross-agency initiatives for capacity building (Bogich et al. 2012). The health resilience research highlights the importance of drawing lessons from others and fostering collaboration with sectors and nations with comparable experiences (Fridell et al. 2020). Multilateral initiatives and global cooperation facilitate the exchange of best practices and medical and human resources, contributing to enhanced recovery from the COVID-19 pandemic (OECD 2020). For instance, during the pandemic, Nepal extensively relied on international actors, notably the United Nations health cluster with national and international representatives, for leading pandemic management, alongside securing crucial supplies, including vaccines, through donations and procurement supported by intergovernmental organisations and foreign governments (MoHP 2022).

The evaluation of collaborative governance on a national level is hindered by challenges, primarily stemming from the absence of tailored indicators and the engagement of diverse stakeholders. While indicators such as the Government Closeness Index (Ivanyna & Shah 2014) and the Civil Society Participation Index (Varieties of Democracy 2020) exist for decentralisation and government-civil society cooperation, researchers often resort to proxy indicators. Guzel, Arslan and Acaravci (2021) employ globalisation index as a proxy for international cooperation and democracy index as a proxy for government-society cooperation, examining their influence on health consequences. In the context of COVID-19, Lupu and Tiganasu (2022) reveal a significant positive association between the globalisation index and the vaccination rates of countries. Furthermore, Sabry (2019) utilises the WGI’s Voice and accountability metric, a measure of democracy, as a proxy for collaborative governance, while Azam et al. (2021) consider democratic accountability as a constituent of institutional quality. Taking the former view, this study utilises the democracy level as one of the indicators of collaborative governance, given that democracies tend to be more conducive to cooperation and collaboration (Bättig & Bernauer 2009; Rey & Barkdull 2005) and are inclined to distribute powers and responsibilities appropriately among the central government, regional and local authorities, and civil society (Hirst 2000).

Considering the findings of studies investigating the relationship between collaborative governance and disaster resilience outcomes, and taking into account proxy indicators of collaborative governance, the following research hypothesis can be formulated:

**Hypothesis 2:** Democracy, decentralisation, civil society engagement, and international cooperation reflect collaborative governance, which exerts a significant positive effect on the COVID-19 resilience outcome.

#### Social capital

Social capital is recognised as an integral aspect of adaptive capacity, enhancing resilience to disaster and climate risks (Kapucu 2012; Paton 2006; Zukowski 2014). According to Paton (2006), the level of adaptive capacity relies on how well public and civic entities empower and involve the public in decision-making. Trust, norms, and networks indicate social capital and play a crucial role in supporting disaster resilience through social support, resource sharing, cooperation, and coordination essential for effective DRM (Aida et al. 2013; Mayunga 2007). Country-specific studies on COVID-19 highlight the role of social capital in fostering heightened concern for others (Makridis & Wu 2021), self-sacrifice for the greater good, resulting in increased compliance with disease control measures (Wu 2021), and voluntary initiatives and collective efforts (Tangcharoensathien et al. 2023).

At the national level, evidence emphasises the crucial role of social capital, specifically trust in government and public engagement, in ensuring an effective response to biological hazards and achieving better resilience outcomes. In the context of COVID-19, social capital has been shown to have a positive impact, contributing to a reduction in the daily number of new cases in Asia (Varkey et al. 2020) and a decrease in excess mortality in seven European countries (Bartscher et al. 2021). Elgar, Stefaniak and Wohl (2020) found that civic involvement and confidence in institutions were linked to lower COVID-19 mortality, potentially by enhancing compliance with COVID-19 directives and guidelines. Furthermore, the credibility of public health agencies and trust are essential, as lacking these elements can amplify the proliferation of infectious diseases (Suk & Semenza 2011). Pandemic control may be hindered by the spread of rumours and misinformation, especially when fuelled by a distrust of government authorities (Madhav et al. 2017). South Korea’s transparent approach to disseminating credible information about COVID-19 strengthened public trust and encouraged people to actively participate in the collective effort to combat the virus (Moon 2020).

Different approaches are used to measure social capital for cross-country disaster research. While some researchers employ a single measure such as voter turnout (e.g. Varkey et al. 2020), others use multiple indicators from macro-level studies such as the World Values Survey (e.g. Elgar et al. 2020) and the Legatum Prosperity Index (e.g. Gömleksiz & Altıntaş 2023; Kapitsinis 2021). Compared to the World Values Survey, the Legatum Prosperity Index developed by the Legatum Institute offers more extensive coverage, including 167 countries in 2020. It identifies five indicators of social capital, including ‘personal and family relationships, social networks, interpersonal trust, institutional trust, and public participation’, each measured by multiple sub-indicators primarily derived from the Gallup World Poll (Legatum Institute Foundation 2020).

Based on the findings from the studies investigating the relationship between social capital and resilience outcomes, and considering indicators of social capital, the following research hypothesis can be formulated:

**Hypothesis 3:** Personal and family relationships, social networks, interpersonal trust, institutional trust, and public participation reflect social capital, which exerts a significant positive effect on the COVID-19 resilience outcome.

## Research methods and design

Following the positivist research approach, this study employs a quantitative method to test the proposed hypotheses. It utilises secondary data and analyses it using the Partial Least Squares Structural Equation Modelling (PLS-SEM) technique with SmartPLS 3 software. The PLS-SEM is chosen for its flexibility in balancing theory and data, making it well-suited for research utilising secondary data, where challenges in revising and refining construct measurements may arise (Nitzl 2016). The study adheres to the procedure outlined by Hair et al. (2022) for PLS-SEM application.

### Sample

The initial pool for sample selection included 195 countries, including all member states of the United Nations and two non-member observer states (Worldometer 2023), highlighting the global impact of the COVID-19 crisis. After excluding countries with significant missing data, the final sample consists of 129 countries. Among these, 48 are classified as high-income, 30 as upper-middle-income, 36 as lower-middle-income, and 15 as low-income. Geographically, the sample spans 34 Asian, 32 African, 37 European, 13 North American, 10 South American, and 3 Oceanian nations.

### Constructs, indicators, and data sources

In this study, the three adaptive capacities – quality of institutions, collaborative governance, and social capital – are modelled as exogenous latent constructs and are hypothesised to influence the endogenous construct – COVID-19 resilience outcome. To account for the heightened vulnerability of the elderly population to COVID-19 (Daoust 2020; Sousa et al. 2020) and the role of coping capacity, particularly economic capacity, in reducing disaster-related deaths (Harlem 2020; Songwathana 2018; Zhang & Huang 2018), the study includes elderly population and national wealth indicator as control variables. The outcome and control variables are single-item constructs, whereas multiple indicators measure the adaptive capacity constructs.

The study adopts an ex-post disaster resilience measurement approach to assess the outcome variable, with a specific emphasis on a country’s capacity to save lives in the face of the pandemic. The COVID-19-related deaths stem not only from the direct impact of COVID-19 infection but also from indirect consequences such as economic downturns, job losses, or constraints on health services. Consequently, the outcome variable (COVID-19 resilience outcome) is quantified in terms of reversed cumulative excess deaths per 100 000 people during COVID-19 as of 31 December 2022, published by The Economist (2023). The estimation of excess deaths compares the total number of deaths during the pandemic period against the anticipated number based on historical data. This measure considers confirmed and unconfirmed COVID-19 deaths, offering insight into the direct and indirect impacts of the crisis on human health and well-being.

Table 1 outlines the study constructs, associated indicators, and the data sources. The exogenous construct dataset predominantly consists of data from 2020 or the nearest available year in cases where 2020 data are unavailable.

**TABLE 1:** Constructs, indicators, measurement, and data source.

Constructs	Indicator	Code	Measurement	Source
Quality of institutions	Government effectiveness	Q1	Government effectiveness index	World Bank (2020b)
	Political stability	Q2	Political stability and absence of violence index	World Bank (2020b)
	Regulatory quality	Q3	Regulatory quality index	World Bank (2020b)
	Control of corruption	Q4	Control of corruption index	World Bank (2020b)
	Rule of law	Q5	Rule of law index	World Bank (2020b)
Collaborative governance	Democracy	C1	Democracy index	Economic Intelligence Unit (2020)
	Decentralisation	C2	Government closeness index	Ivanyna and Shah (2014)
	Civil society engagement	C3	Civil society participation index	Varieties of Democracy (2020)
	International cooperation	C4	Political globalisation index	Gygli et al. (2019)
Social capital	Personal and family relationships	S1	Personal and family relationships	Legatum Institute Foundation (2020)
	Social networks	S2	Social networks	Legatum Institute Foundation (2020)
	Interpersonal trust	S3	Interpersonal trust	Legatum Institute Foundation (2020)
	Institutional trust	S4	Institutional trust	Legatum Institute Foundation (2020)
	Public participation	S5	Civic and social participation	Legatum Institute Foundation (2020)
Elderly population	Elderly population	Elderly	Proportion of the population over 65 years of age	World Bank (2020a)
Wealth	GDP per capita	GDP	GDP per capita, PPP (2020)	World Bank (2022)
COVID-19 resilience	Reversed excess deaths	Rev_ExD	−1 * (cumulative excess deaths per 100 000 population as of 31 December 2022)	The Economist (2023)

GDP, Gross domestic product; PPP, current international $.

## Results

### Data preparation

After gathering data from 195 countries, the dataset underwent processing using Statistical Package for the Social Sciences (SPSS). Because of the absence of data on three indicators of collaborative governance in 66 countries, these countries were eliminated, resulting in a refined dataset of 129 countries. The remaining missing values were addressed using the mean substitution method in accordance with the guidelines by Hair et al. (2022), who suggest multiple methods for handling missing data in datasets with less than 5% missing values per indicator. Descriptive statistics were then used to assess indicator distribution and identify outliers.

Subsequently, skewness and kurtosis assessments were performed, and variables falling outside the acceptable range of –2 to +2 underwent transformations. Decentralisation, interpersonal trust, and gross domestic product (GDP) per capita were subjected to a base-10 logarithmic transformation; social networks was squared, and reversed excess death underwent a logarithmic transformation using the formula log (100 + reversed excess death). Outliers were identified using box plots and replaced with the closest non-extreme values. Additionally, the polarity of the outcome variable, measured as excess deaths per 100 000 population, was changed from negative to positive by multiplying the variable by –1. Finally, all indicators were standardised into *z*-scores for analysis.

### Measurement model

**FIGURE 1:**
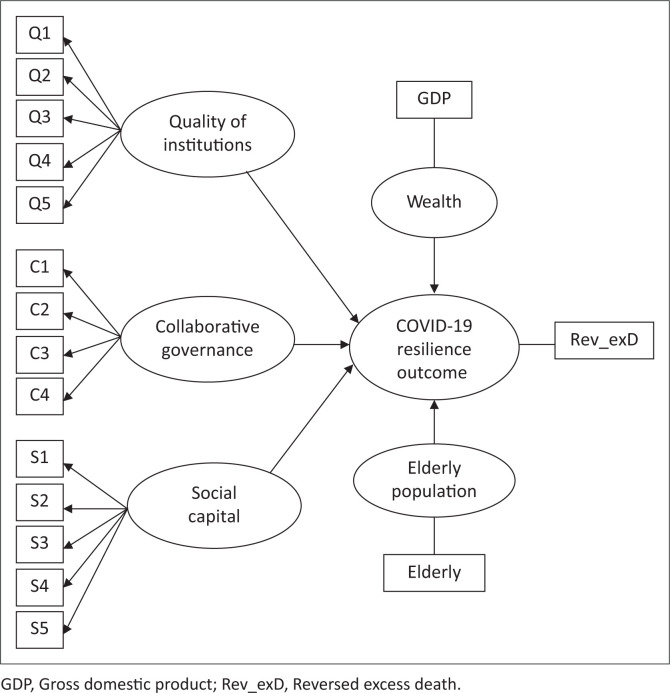
Measurement model.

The measurement model illustrates how constructs relate to their indicators in Structural Equation Modelling (SEM), considering whether they will be modelled as formative or reflective. A reflective construct is a trait describing its indicators, while a formative construct is a combination of its indicators (Hair et al. 2022). The PLS-SEM literature lacks a well-defined approach to modelling national-level indicators utilising secondary data. In a cross-country study, Ngobo and Fouda (2012) model good governance as a reflective construct, citing the high correlation between the WGI indicators as a challenge for formative modelling. Another cross-country study, Hanafiah and Zulkifly (2019), treats constructs measured with soft data as reflective and hard data as formative. Aligned with these studies and guided by Hair et al. (2022), the three adaptive capacity constructs are modelled as reflective constructs in this study, as depicted in Figure 1.

### Measurement model assessment

The measurement model was assessed on the basis of indicator reliability, internal consistency reliability, convergent validity, and discriminant validity (Hair et al. 2022), as presented in Table 2.

**TABLE 2a:** Measurement model assessment results.

Construct	Item	Outer loading (significance)	Cronbach’s alpha	Composite reliability	AVE
Quality of institutions	Q1	0.934 (0.000)	0.975	0.963	0.839
	Q2	0.751 (0.000)	0.975	0.963	0.839
	Q3	0.913 (0.000)	0.975	0.963	0.839
	Q4	0.992 (0.000)	0.975	0.963	0.839
	Q5	0.971 (0.000)	0.975	0.963	0.839
Collaborative governance	C1	0.679 (0.000)	0.820	0.841	0.582
	C2	0.921 (0.000)	0.820	0.841	0.582
	C3	0.502 (0.003)	0.820	0.841	0.582
	C4	0.875 (0.000)	0.820	0.841	0.582
Social capital	S3	0.737 (0.000)	0.665	0.817	0.598
	S4	0.764 (0.000)	0.665	0.817	0.598
	S5	0.678 (0.000)	0.665	0.817	0.598

AVE, average variance extracted.

**TABLE 2b:** Measurement model assessment results.

Construct	Heterotrait-monotrait estimates			
	Quality of institutions	Collaborative governance	Social capital	Elderly population
Quality of institutions	-	-	-	-
Collaborative governance	0.779	-	-	-
Social capital	0.619	0.314	-	-
Elderly population	0.703	0.853	0.063	-
Wealth	0.818	0.642	0.270	0.708

Indicator reliability is indicated by the size of outer loadings, with higher loadings (0.708 or higher) suggesting significant commonality among indicators. According to Hair et al. (2022), indicators with outer loadings less than 0.40 ought to be eliminated. The results from the bootstrapping procedure involving 10 000 samples revealed that indicators S1 and S2 had outer loadings less than 0.40 and negative loadings, leading to their exclusion from the model.

Internal consistency reliability of the reflective constructs was evaluated using Cronbach’s alpha and composite reliability. In exploratory research, values within the range of 0.60–0.70 meet acceptable standards (Hair et al. 2022). The results indicated that both Cronbach’s alpha and composite reliability exceeded 0.60 for all three adaptive capacity constructs.

Convergent validity, indicating how well a construct effectively explains the variance among its items (Hair et al. 2022), was evaluated through average variance extracted (AVE) estimates. The results demonstrated that AVE values for all three adaptive capacity constructs surpassed 0.50, aligning with the convergent validity criteria proposed by Hair et al. (2022).

Finally, discriminant validity, which assesses how distinct a construct is from others, was evaluated using heterotrait-monotrait (HTMT). Typically, a value above 0.90 implies a lack of discriminant validity, but for conceptually distinct constructs, a 0.86 threshold is considered (Hair et al. 2022). The results demonstrated that HTMT estimates between constructs were below 0.86, indicating satisfactory discriminant validity.

### Structural model assessment

In the first step, collinearity was examined using inner variance inflation factor (VIF) values from the PLS algorithm. The results (see Table 3) reveal that all predictor constructs have VIF values below the threshold of 5.0, suggesting that there are no critical collinearity problems (Hair et al. 2022).

**TABLE 3:** Partial least squares structural equation modelling results.

Hypothesis	Path	Path coefficient & *P*	VIF	*f* ^2^	Decision
H1:	Quality of institutions → COVID-19 resilience outcome	0.520 (0.033*)	4.51	0.127	Accepted
H2:	Collaborative governance → COVID-19 resilience outcome	−0.229 (0.036*)	2.62	0.042	Rejected
H3:	Social capital → COVID-19 resilience outcome	0.323 (0.002**)	2.07	0.107	Accepted
	Elderly → COVID-19 resilience outcome	−0.403 (0.002**)	3.65	0.094	-
	Wealth → COVID-19 resilience outcome	−0.259 (0.083)	2.89	0.049	-
	*R*^2^ = 0.507				
	*Q*^2^ = 0.452				

Note:

* *p* < 0.05;

** *p* < 0.01.

VIF, Variance Inflation Factor.

Path coefficients and standard errors for the hypothesised relationships were derived using bootstrapping with 10 000 samples to evaluate the significance and relevance of the relationships within the structural model. As shown in Table 3, the results indicate that quality of institutions (*p* < 0.05) and social capital (*p* < 0.01) exhibit significant positive associations with COVID-19 resilience outcome. In contrast, collaborative governance surprisingly exhibits a significant negative association with COVID-19 resilience outcome (*p* < 0.05).

The models’ explanatory power was evaluated using the coefficient of determination (*R*^2^), which signifies the collective impact of exogenous constructs on the endogenous construct. While acceptable *R*^2^ values vary by context (Hair et al. 2022), benchmarks of 0.75, 0.50, and 0.25 are deemed significant, moderate, and weak explanatory power, respectively (Hair et al. 2019). With an adjusted *R*^2^ value of 0.507, the model demonstrates moderate explanatory power in explaining COVID-19 resilience outcome.

The *f*^2^ value (effect size) indicates the change in the *R*^2^ when a particular predecessor construct is removed from the model (Hair et al. 2022). Cohen (1988) classifies effect sizes as small, medium, and large when the *f*^2^ values exceed 0.02, 0.15, and 0.35, respectively. The results indicate that all three adaptive capacity constructs exhibit small effect sizes, falling within the 0.02–0.15 range.

Subsequently, the *Q*^2^ metric, derived from the blindfolding procedure, was used to assess the predictive accuracy of the endogenous construct. A *Q*^2^ value greater than zero suggests predictive accuracy, with values above 0, 0.25, and 0.50 denoting small, medium, and large predictive relevance, respectively (Hair et al. 2019). With a *Q*^2^ value of 0.452, the results suggest that the model demonstrates predictive accuracy with a medium level of predictive relevance.

Lastly, control variables were examined, revealing a significant negative association between the elderly population (elderly) and the outcome variable. Although not statistically significant, wealth demonstrated a negative linear relationship with COVID-19 resilience outcome, suggesting that higher GDP per capita is associated with reduced disaster resilience. Cao et al. (2021) and Varkey et al. (2020), who found that higher GDP per capita correlates with greater impacts of COVID-19, contend that wealthier countries, which typically have more economic activities and social interactions, are more susceptible to the spread of COVID-19. Thus, it is likely that economic capacity functioned as a vulnerability determinant, exacerbating viral transmission rates and thereby contributing to elevated COVID-19-related mortality.

## Discussion

Through a quantitative analysis of secondary data from 129 countries and the application of PLS-SEM, this study investigated whether adaptive capacities – namely, the quality of institutions, collaborative governance, and social capital – determine the COVID-19 resilience outcome, measured by countries’ ability to minimise both direct and indirect deaths associated with the crisis. The study model revealed a statistically significant and positive impact of institutional quality on the resilience outcome. This association is in line with the findings of Serikbayeva, Abdulla and Oskenbayev (2021) and Liang et al. (2020), establishing a substantial link between government effectiveness and a reduction in COVID-19 fatalities. The positive influence of institutional quality on resilience manifests in diverse ways. Nabin et al. (2021) and Coccia (2021) demonstrate a significant positive association between higher institutional quality and increased vaccination rates. Research by Bunyavejchewin and Sirichuanjun (2021) posits that factors such as the rule of law and regulatory quality influence a nation’s financial responses to counter the adverse impacts of the pandemic. These findings suggest that countries with higher-quality institutions can effectively implement and enforce relevant policy measures, mitigating both direct and indirect impacts of the pandemic and contributing to resilient outcomes.

Unexpectedly, the study found a significant negative association between collaborative governance and COVID-19 resilience. Country-specific studies, such as Choi’s (2020) analysis of South Korea, Huang’s (2020) analysis of Taiwan, and Criado and Guevara-Gómez’s (2021)’s analysis of Spain, emphasise the significant impact of collaborative governance in enhancing the COVID-19 response. In contrast, the findings from this research indicate that collaborative governance may have counterproductive effects when assessed in conjunction with diverse influencing factors and conducted at a broader level encompassing various countries. Challenges inherent in collaborative governance during the policy process may explain its role in impeding disaster-resilient outcomes. Ansell and Gash (2008) identified several challenges in implementing collaborative governance, including time-consuming trust-building, insufficient commitment from public agencies, policy manipulation by powerful stakeholders, consensus-building difficulties, and distrust among partners. These challenges may be particularly heightened during the disaster response when prompt decision-making and coordinated efforts are crucial. Importantly, establishing trust and nurturing relationships among key stakeholders in the pre-disaster phase becomes pivotal to ensuring coordinated efforts during the post-disaster phase (Kapucu 2008; Pratama & Nurmandi 2020).

Finally, this study conclusively established a significant positive influence of social capital on the COVID-19 resilience outcome. This aligns with the perspective that social capital functions as a valuable asset, fostering cooperation and collective action for mutual benefits (Bhandari & Yasunobu 2009; Putnam 1993). The significant role of social capital in mitigating the negative impacts of the pandemic is highlighted in both cross-country studies (e.g. Elgar et al. 2020; Varkey et al. 2020) and country-specific studies (e.g. Carter & Cordero 2022; Wilson et al. 2023; Wu 2021).

While there is general agreement on the significant impact of social capital on pandemic outcomes in statistical research focussed on causal inference, the specific reasons behind its mitigating effect remain unclear (Makridis & Wu 2021). However, insights from some studies underscore the pivotal role of social capital in potentially alleviating both the direct and indirect impacts of the pandemic, thereby contributing to resilient outcomes. Its influence in promoting better compliance with pandemic measures (Wu 2021) implies a potential reduction in virus spread and, consequently, a decline in direct deaths. Additionally, its ability to facilitate job access through social networks (Samutachak et al. 2023) and provide access to food and other necessities for vulnerable groups (Center for Philanthropy and Civil Society 2021) suggests a potential mitigation of indirect economic impacts. This, in turn, may influence indirect deaths linked to job loss, poverty, or unsafe employment conditions.

While this study considered social capital as a latent construct manifesting in various forms, multiple studies emphasise the diverse ways in which different forms contribute to varied outcomes during the COVID-19 pandemic (Makridis & Wu 2021; Wu 2021). For example, Wu (2021) observed that trust in government produced more favourable outcomes than social trust in China. In Malaysia, Said et al. (2022) identified positive impacts of individual social capital, encompassing attributes such as reciprocity, trustworthiness, attachment, and cooperation, on mental health during the pandemic. Furthermore, research by Samutachak et al. (2023) underscored the critical role of bonding social capital within families or communities, with the bridging dimension coming into play when depleted, connecting individuals with resources beyond their immediate network in Thailand. This underscores the idea that different dimensions and forms of social capital contribute synergistically to achieve resilient outcomes.

## Conclusion

This study aimed to contribute to disaster research by operationalising and empirically investigating the effects of three adaptive capacities – institutional quality, collaborative governance, and social capital – on COVID-19 resilience outcomes. After controlling for the elderly population and GDP per capita, the study identified significant positive effects of institutional quality and social capital on the resilience outcome. The crucial implication of this research is that a targeted emphasis on enhancing institutional quality and social capital is essential, particularly given the uncertainties in contemporary interconnected and complex societies. These capacities play a critical role in mitigating both the direct and indirect impacts of the pandemic by fostering societal adaptability to evolving needs, ultimately contributing to resilient outcomes.

While the primary focus of this study centred on post-disaster contexts and assessing adaptive capacity for responding to unforeseen impacts, the identified capabilities remain relevant for both pre- and post-disaster scenarios. For instance, Makridis and Wu (2021) argue that communities with high social capital exhibit improved economic performance and healthier people in the pre-disaster context, leading to enhanced responses during disease outbreaks. In addition, Oppenheim et al. (2019) argue that epidemic preparedness, which includes the ability to detect potential threats and prepare for effective response, heavily relies on the capacity of government institutions. Thus, adopting a long-term perspective on disaster resilience by recognising the significance of institutional and social forces is crucial, as these forces can influence DRM efforts both before and after a disaster.

However, the study revealed a significant negative association between collaborative governance and resilience outcomes, highlighting the need for more nuanced approaches to understand this relationship. This research advocates for adopting a disaggregated approach to unravel the role of collaborative governance in DRM and overall resilience. This approach may entail assessing the individual contributions of each stakeholder to collaborative efforts, identifying the combinations of stakeholders that yield optimal results during specific phases of disasters, and evaluating key attributes such as levels of trust and commitment among stakeholders. By doing so, we can determine the optimal levels of collaborative governance needed for each phase to enhance overall disaster resilience. This refined understanding holds the potential to significantly contribute to the development of targeted strategies for building resilience.
